# Identification of *WRKY* Gene Family from *Dimocarpus longan* and Its Expression Analysis during Flower Induction and Abiotic Stress Responses

**DOI:** 10.3390/ijms19082169

**Published:** 2018-07-25

**Authors:** Dengwei Jue, Xuelian Sang, Liqin Liu, Bo Shu, Yicheng Wang, Chengming Liu, Jianghui Xie, Shengyou Shi

**Affiliations:** 1 Key Laboratory of Tropical Fruit Biology (Ministry of Agriculture), South Subtropical Crops Research Institute, Chinese Academy of Tropical Agricultural Sciences, Zhanjiang 524091, China; juedengwei@126.com (D.J.); anyue1220@126.com (X.S.); lolitallq@163.com (L.L.); bshbest@163.com (B.S.); ychw08@163.com (Y.W.); 2 College of Horticulture, South China Agricultural University, Guangzhou 510642, China; cmliu@scau.edu.cn

**Keywords:** longan, WRKY, expression analysis, flower induction, abiotic stress

## Abstract

Longan is an important fruit tree in the subtropical region of Southeast Asia and Australia. However, its blooming and its yield are susceptible to stresses such as droughts, high salinity, and high and low temperature. To date, the molecular mechanisms of abiotic stress tolerance and flower induction in longan have not been elucidated. WRKY transcription factors (TFs), which have been studied in various plant species, play important regulatory roles in plant growth, development, and responses to stresses. However, there is no report about WRKYs in longan. In this study, we identified 55 *WRKY* genes with the conserved WRKY domain and zinc finger motif in the longan genome. Based on the structural features of WRKY proteins and topology of the phylogenetic tree, the longan WRKY (DlWRKY) family was classified into three major groups (I–III) and five subgroups (IIa–IIe) in group II. Tissue expression analysis showed that 25 *DlWRKYs* were highly expressed in almost all organs, suggesting that these genes may be important for plant growth and organ development in longan. Comparative RNA-seq and qRT-PCR-based gene expression analysis revealed that 18 *DlWRKY* genes showed a specific expression during three stages of flower induction in “Sijimi” (“SJ”), which exhibited the “perpetual flowering” (PF) habit, indicating that these 18 *DlWRKY* genes may be involved in the flower induction and the genetic control of the perpetual flowering trait in longan. Furthermore, the RT-qPCR analysis illustrated the significant variation of 27, 18, 15, 17, 27, and 23 *DlWRKY* genes under SA (Salicylic acid), MeJA (Methyl Jasmonate), heat, cold, drought, or high salinity treatment, respectively, implicating that they might be stress- or hormone-responsive genes. In summary, we systematically and comprehensively analyzed the structure, evolution, and expression pattern of the *DlWRKY* genes. The results presented here increase our understanding of the WRKY family in fruit trees and provide a basis for the further elucidation of the biological function of *DlWRKY* genes in longan.

## 1. Introduction

Longan (*Dimocarpuslongan* Lour.) is an important subtropical fruit tree in the family Sapindaceae, which is grown in many subtropical and tropical countries with most of the production in Southeast Asia and Australia [1]. Biennial bearing is the most serious problem that affects longan fruit products. Among the factors that affect *D. longan* fruit yield, the difficulty and unstableness to blossom is one of the most challenging problems [2]. Floral bud induction of *D. longan* requires favorable conditions such as a period of low temperature (vernalization), suitable salinity, and dry conditions. To obtain a stable high yield, off-season flowering in longan is achieved by chemical treatment with potassium chlorate (KClO_3_) application [3,4]. Nevertheless, the induction effect varies in different regions and varieties. Therefore, the study of the molecular regulatory mechanisms of flower induction and abiotic stress tolerance in longan is particularly important for understanding and solving the problems associated with fruit yield. However, due to the long generation time and lack of genome information, knowledge of the molecular regulatory mechanisms of flower induction and abiotic stress tolerance in longan is scarce.

As an important developmental process in the plant life cycle, flowering is directly linked to production whenever seeds or fruits are harvested [5]. The molecular and genetic bases of flowering have been well studied in *Arabidopsis thaliana* [6,7,8]. There are at least five major flowering pathways in *Arabidopsis*, including the photoperiod, autonomous, vernalization, gibberellin (GA), and aging pathways [9]. These pathways activate or inhibit floral transformation through a series of flower integron genes, such as the flowering locus T (*FT*), flowering locus C (*FLC*), and constans (*CO*) [10]. In addition, several transcription factors (TFs), such as MADS-domain TFs [11], NACs [12], MYBs [13], and DREBs [14], participate in the signaling of flowering regulation. As the seventh largest TF family in flowering plants, many WRKY genes are also involved in the determination of flowering time [15]. For example, in *A*. *thaliana*, the lines over-express *GsWRKY20*, *MlWRKY12*, and *WRKY71* in the flowers earlier than in the wild-type [16,17,18]. A recent research study found that two WRKY proteins (AtWRKY12 and AtWRKY13) played opposite functions in controlling the flowering time under short-day conditions in *A*. *thaliana* partly through mediating the effect of GA_3_. The *wrky*12 mutant exhibits late flowering and the *wrky*13 mutant shows earlier flowering than that of the wild-type [19].

Abiotic stresses such as drought, heat, salt, and cold are the major causes of declined crop productivity worldwide. At the molecular level, several TFs, such as AP2/EREBP, NAC, WRKY, bZIP, MYB, and bHLH play a vital role in regulating downstream genes to protect plants from these stresses [20]. As one of the largest TF families in plants, the WRKY TFs also play pivotal roles in regulating many abiotic stress reactions [15]. In *Arabidopsis*, some of the *AtWRKYs* respond strongly to various abiotic stresses, such as salinity, drought, and cold [21,22,23,24]. In rice, 11 *OsWRKY* genes showed variable responses to salt, polyethylene glycol (PEG), and cold or heat stresses [25]. Overexpression of *OsWRKY47* increased both the drought tolerance and yield compared with wild-type plants [26]. In mulberry, *Morus013217* and *Morus002784* show high accumulation in response to cold and salt stresses. *Morus005757* shows significant up-regulation in response to dehydration stress, salinity stress, and SA and ABA (Abscisic acid) treatments [27]. Similar results were also found in wheat, common bean [28], grape [29], pineapple [30], soybean [31], moso bamboo [32], *Caragana intermedia* [33], peanut [34], and broomcorn millet [35]. These observations suggest that studying the WRKY gene families may provide valuable insights into the mechanism underlying abiotic stress tolerance in plants. As perennials growing in the subtropical and tropical area, some abiotic stresses, such as drought, heat, salt, and cold often have an adverse effect on the growth and yield of longan. However, given the lack of genome information, the identified and functions of *WRKY* genes in longan are still unknown.

In the present study, we performed a genome-wide identification of WRKY TFs in longan and analyzed their gene structures, conserved motifs, and expression patterns in nine different tissues. This work also determined the expression profiles of longan WRKY (*DlWRKY*) in three flowering stages of two longan cultivars and measured their transcript abundance in response to different phytohormone treatments and various abiotic stresses. This study provides a basis for future studies on *DlWRKY* gene family evolution and function.

## 2. Results

### 2.1. Identification of WRKY Gene Family in Longan

To extensively identify the WRKY genes in longan, whole-genome scanning was used to identify the genes which contain the particular domain by both the hidden Markov model (HMM) and Blastn search methods. In total, 59 candidate WRKY genes were identified (Table S1). After the WRKY domain scanning and sequence alignment, three genes (*Dlo_*007676.1, *Dlo_*032703.1, and *Dlo_*028398.1) without a complete predicted WRKY domain and one redundant gene (*Dlo_*037584.1) were removed. Finally, 55 *DlWRKY* genes were determined in the longan genome (Table 1). According to their chromosome locations, the 55 *DlWRKY* genes were designated *DlWRKY*1*–DlWRKY*55. In addition, the basic properties of *DlWRKY* genes, including the length of the full-length sequence, open reading frame (ORF), protein sequence, molecular weight (MW), and PI, were systematically evaluated (Table 1). The average length of these *DlWRKY* genes was 2417 bp and the length mainly centered on the range of 892 bp (*DlWRKY*12) to 5385 bp (*DlWRKY*36). Meanwhile, the length of the ORF was mainly distributed from 480 bp (*DlWRKY*12 and *DlWRKY*34) to 3813 bp (*DlWRKY*36), with an average of 1237 bp. The length of the protein sequences ranged from 160 AA (*DlWRKY*12 and *DlWRKY*34) to 1271 AA (*DlWRKY*36), with an average of 411 AA. The protein MW ranged from 18.10 kDa (*DlWRKY*34) to 143.77 kDa (*DlWRKY*36), with an average of 44.73 kDa. The predicted isoelectric point of the DlWRKY proteins varied from 4.62 (*DlWRKY*22) to 9.77 (*DlWRKY*13), with an average of 7.11.

### 2.2. Phylogenetic Analysis of DlWRKY

**Figure 1:**
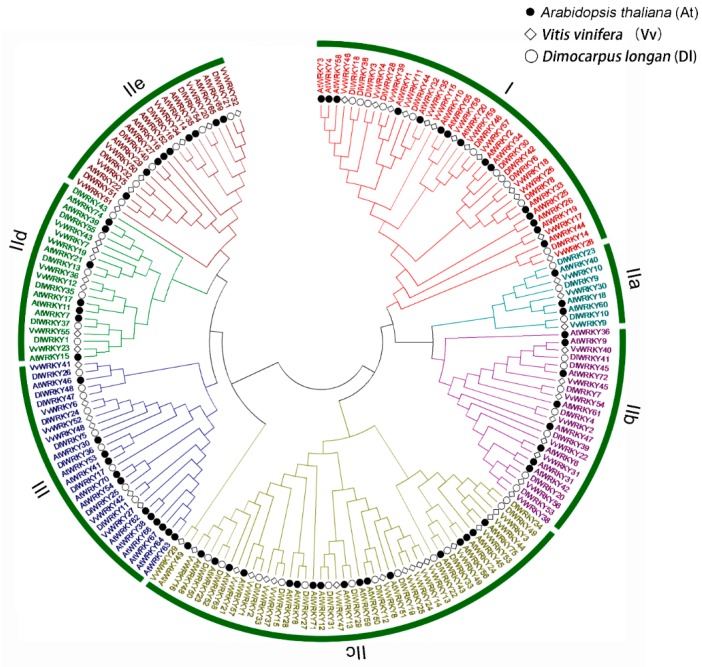
The phylogenetic analysis of the longan WRKY proteins with orthologous members from grape and *Arabidopsis.* The maximum likelihood phylogenetic tree was constructed by MEGA 6.0. Different groups of DlWRKY proteins are indicated by a circle and the different colors.

A phylogenetic tree was constructed using the maximum likelihood (ML) method and based on multiple alignments of longan, grape, and *Arabidopsis* WRKY domain aa sequences. As shown in Figure 1, the phylogenetic results revealed that all the DlWRKY proteins could be categorized into three groups (I, II, and III). Eleven DlWRKY proteins were considered to be group I, which included two WRKY domains and a C_2_H_2_ (C–X_4_–C–X_22–23_–HXH) zinc finger motif. A total of 35 DlWRKY proteins contained one WRKY domain and a C_2_H_2_ (C–X_4–5_–C–X_23_–HXH) zinc-binding motif, which were classified as group II. The nine remaining genes were assigned to Group III, which consisted of a single WRKY domain and a C_2_CH (C–X_7_–C–X_23_–HXC) zinc-binding motif. According to the WRKY subgroup classification of *Arabidopsis*, the DlWRKYs in Group II were further subdivided into five subgroups, including groups IIa (3), IIb (7) IIc (13), IId (6), and IIe (6).

### 2.3. Multiple Sequence Alignment and Structure Analysis

The WRKYGQK sequence is a considerably conservative motif of WRKY proteins and several variants of this conserved WRKY motif have been reported in plants [36], including WRKYGEK, WRKYGKK, WSKYEQK, and WRKYSEK. In the present study, this motif was observed in all longan WRKY proteins and three variants of this motif were also found. The majority of DlWRKY proteins contained the WRKYGQK motif, and WRKYGKK and WKKYRQK were observed in DlWRKY19 and DlWRKY47, respectively. The other remarkably conservative motif was a zinc finger structure which contained two types of zinc finger motifs: C–X_4-5_–C–X_22–23_–HXH and C–X_7_–C–X_23_–HXC. A total of 46 DlWRKY proteins contained C–X_4-5_–C–X_22–23_–HXH, and nine DlWRKY proteins contained C–X_7_–C–X_23_–HXC, which all belonged to Group III (Table S1).

**Figure 2:**
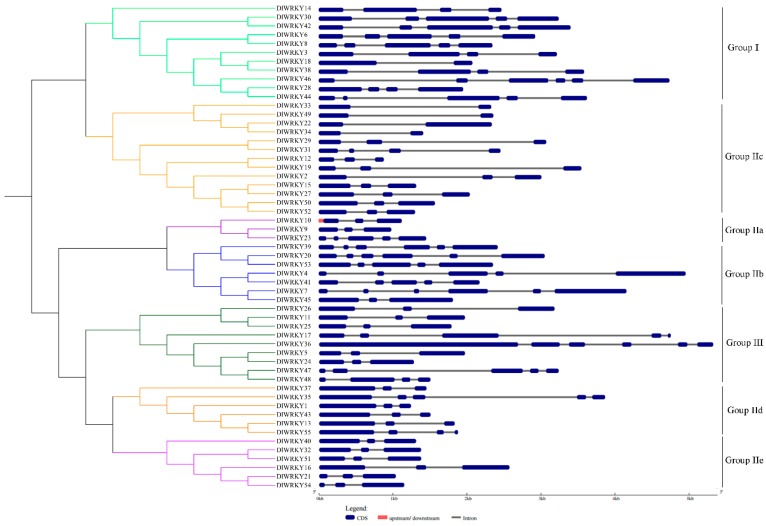
The unrooted phylogenetic tree (**left**) and gene structure (**right**) of 55 DlWRKY proteins. The phylogenetic tree was constructed by MEGA 6.0. The red color indicates the untranslated 5′- and 3′-regions; the blue color indicates exons; and the gray color indicates introns.

According to the Gene Structure Display Server (GSDS) website, the number of introns was in the range of 1–5 in all the longan WRKY gene families, with most of *DlWRKY* genes containing 2–4 introns (*n* = 81.0%). The average number of introns was 2.82. In addition, the phylogenetic analysis of the *DlWRKY* gene family showed that the genes within the same group generally exhibited a similar exon/intron structure. For example, subgroup IIe contained two introns (Figure 2).

**Figure 3:**
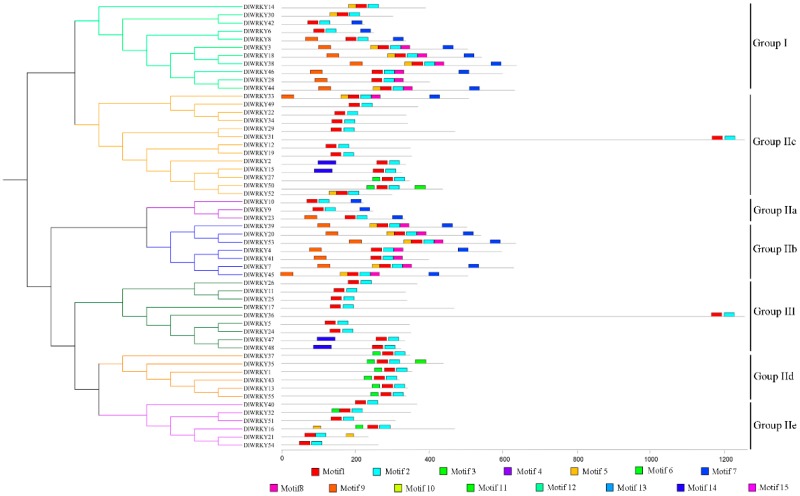
The unrooted phylogenetic tree (**left**) and conserved motifs (**right**) of 55 DlWRKY proteins. The phylogenetic tree was constructed using the same method used in Figure 2. Different colors represent various groups. MEME was used to predict motifs, and these motifs are represented by boxes.

To further understand the similarity and diversity of motif composition among different DlWRKY proteins, a phylogenetic tree based on the full-length DlWRKY proteins was constructed (Figure 3). The motifs in the DlWRKY protein sequences were also predicted using MEME (http://meme.sdsc.edu/meme/cgi-bin/meme.cgi) (Figure 3 and Table S2). A total of 15 motifs were identified to illustrate the WRKY protein structure in longan. The results showed that the number of motifs in DlWRKYs ranged from 2 to 6, and the length of motifs ranged from 21 to 50 amino acids. Among the 15 identified motifs, motifs 1 and 2, characterized as WRKY domains, were broadly distributed across the DlWRKYs.

### 2.4. Tissue-Specific Expression Patterns of DlWRKY

**Figure 4:**
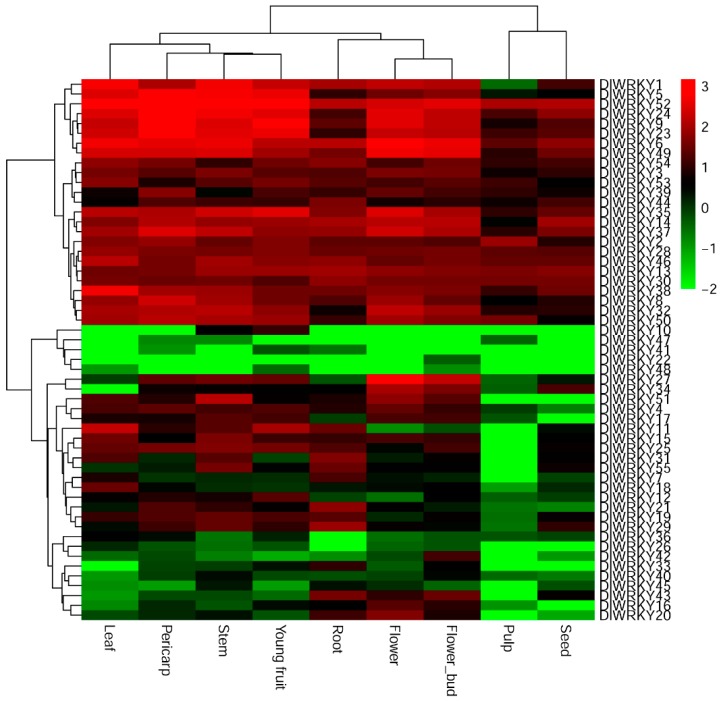
The heat map of the *DlWRKY* gene expression profiles in different tissues. The color scale represents the log_10_ expression values; the red and green colors indicate the higher or lower transcript abundances compared to the relevant control, respectively.

To generate expression profiles of *DlWRKY* genes under normal conditions, the expression levels of the 55 *DlWRKY* genes in the root, stem, leaf, seed, young fruit, pulp, pericarp, flower, and flower bud were investigated by the RNA-seq analysis. The log_10_ (FPKM + 0.01) values of the transcripts were clustered hierarchically and displayed in a heat map (Figure 4 and Table S3). The results showed that 96.36% (53 of 55) of *DlWRKYs* were expressed in young fruits and 94.55% were expressed in the pericarp, stems, and flower bud. A total of 90.91%, 89.09%, and 81.82% of *DlWRKYs* were expressed in the flower, leaf, root, and seed, respectively. Only a few *DlWRKY* genes were detected in pulps (67.27%). Approximately 60% (33 of 55) of the *DlWRKY* genes were expressed in each tested tissue, in which 25 *DlWRKY* genes (*DlWRKY*1, 2, 3, 5, 6, 8, 9, 13, 14, 23, 24, 28, 30, 32, 35, 37, 38, 39, 44, 49, 50, 52, 53, and 54) were highly expressed in at least six longan tissues. In contrast, 12 *DlWRKY* genes *(DlWRKY*10, 12, 18, 22, 26, 36, 40, 41, 42, 45, 47, and 48) were expressed at low levels in all tested tissues. Furthermore, *DlWRKY*22 only displayed a significantly low expression in the flower bud. *DlWRKY*10, 22, 41, 47, and 48 were preferential accumulation in two or three tissues.

### 2.5. Comparative Expression Profiles of Two Longan Species during the Flowering Process

**Figure 5:**
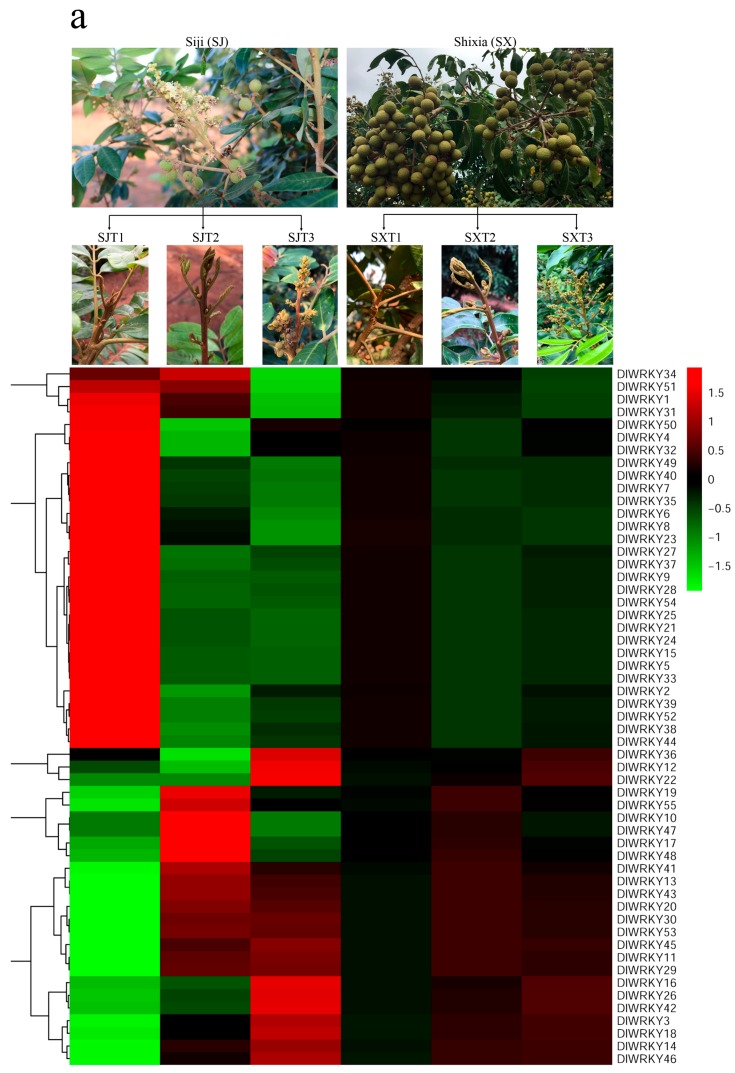

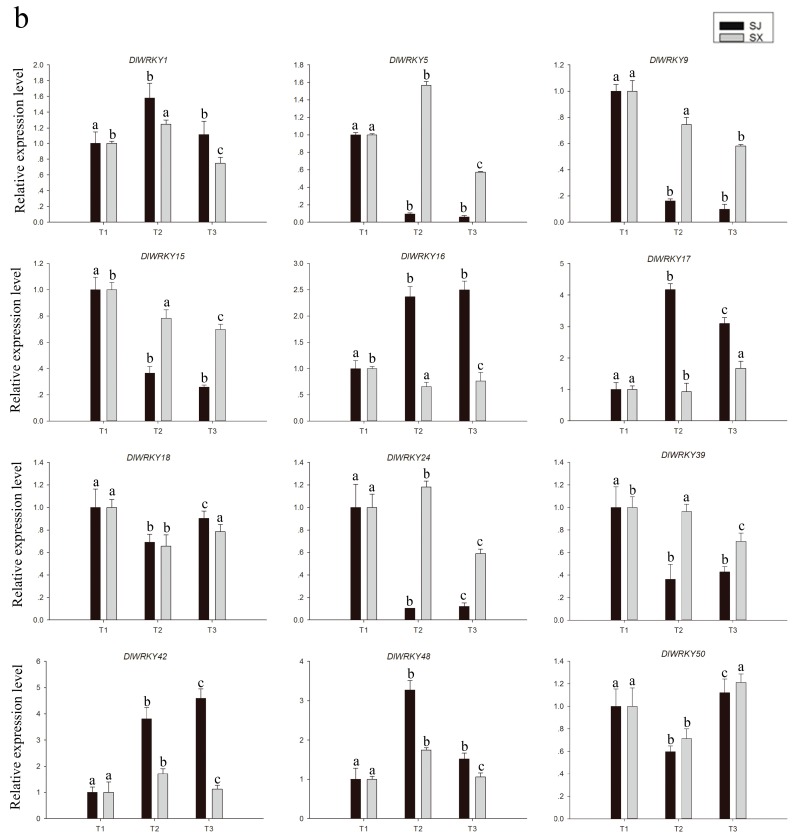
The expression profiles of *DlWRKY* in two longan species during the floral induction process. (**a**) A heat map showing the comparative expression level of the *WRKY* genes in the three flowering stages of“SJ” and “SX”. The color scale represents the log_10_ expression values. Genes with comparatively low expression values are shown using shades of green, and high expression values are represented using shades of red. The three flowering stages of SJ are indicated by SJT1, SJT2, and SJT3. The three flowering stages of SX are indicated by SXT1, SXT2, and SXT3. (**b**) Relative expression levels of the twelve *DlWRKYs* during the three flowering stages of the two longan species by qRT-PCR. For each gene, the relative expression level in T1 (dormant apical bud) was set as one, and the longan *actin* gene was used as the internal expression control. The data represent the mean ± SD of the three replicates. Values with the same letter were not significantly different when assessed using Duncan’s multiple range test (*p* < 0.05, *n* = 3).

Although the involvement of many WRKY genes has been examined in the control of flowering time [15], the expression of *DlWRKY* genes during flower induction has not been studied extensively. In the present study, we also analyzed the expression patterns of 55 *DlWRKY* genes in two longan species during the three flowering stages by RNA-seq analysis (Table S4). Heat maps were constructed based on the log_10_ (FPKM + 0.01) values for the 55 *DlWRKY* genes (Figure 5a). Based on the criteria for *p*-values <0.05 and fold changes ≥2, the *DlWRKY* genes that were differentially expressed during the three flowering stages of the two longan species were identified. Interestingly, the results showed that all 55 *DlWRKY* genes were constructively expressed in the three test flowering stages of the “SX” longan, while 18 *DlWRKY* genes showed a specific expression in the “SJ” longan. Among the 18 *DlWRKY* genes, 12 (*DlWRKY*5, 7, 8, 9, 15, 21, 23, 24, 25, 39, 52, and 54) showed a continuously down-regulated expression through the three flowering stages, and four genes (*DlWRKY*16, 17, 41, and 42) showed an up-regulated expression. Moreover, two genes (*DlWRKY*10 and 48) showed a transient up-regulation at the second stage and a down-regulation at the third stage.

To validate the expression levels obtained from the RNA-seq data, twelve *DlWRKY* genes (*DlWRKY*1, 5, 9, 15, 16, 17,18,24,39,42, 48, and 50) were selected from the six different longan WRKY groups for the quantitative real-time reverse transcription polymerase chain reaction (qRT-PCR) analysis. Consistent with the result of the RNA-seq analysis, the transcript levels of all twelve *DlWRKY* genes did not exhibit any significant differences in the “SX” longan between the three flowering stages (Figure 5b). In addition, the relative expression level of *DlWRKY*1, *DlWRKY*18, and *DlWRKY*50 did not exhibit any significant differences in ‘"“SJ” during the three flowering stages. The expression levels of *DlWRKY*16, 17, 42, and 48were up-regulated in the second and third stage. The transcript level of *DlWRKY*5, 9, 15, 24, and *DlWRKY*39 was down-regulated in the second and third stages (Figure 5b). In general, the expression levels obtained by qRT-PCR for these genes are similar to the results obtained from the RNA-seq data.

### 2.6. Differential Expression of DlWRKY Genes in Response to Stress and Hormonal Treatments

**Figure 6:**
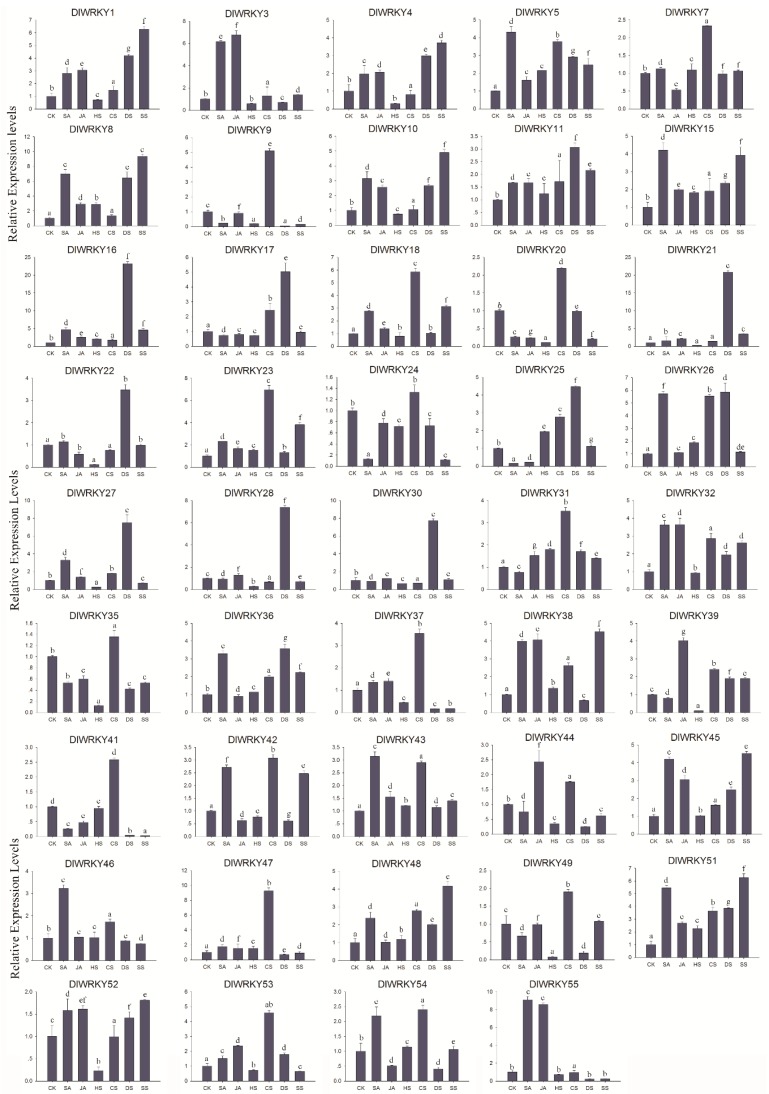
The expression patterns of the selected *DlWRKY* genes under various hormonal and abiotic stresses. The x-axis indicates various treatments and the y-axis indicates the relative expression level. Error bars were obtained from three independent biological replicates. Values with the same letter were not significantly different when assessed using Duncan’s multiple range test (*p* < 0.05, *n* = 3). SA represents salicylic acid, JA represents jasmonic acid, HS represents heat stress, CS represents cold stress, DS represents drought stress, and SS represents salinity stress.

The expression patterns of 55 *DlWRKY* genes were investigated in response to hormonal and various stresses by using qRT-PCR. As shown in Figure 6 and Figure S1, the majority of the *DlWRKY* genes (44 of 55) were up-regulated or down-regulated by >2-flod under at least one tested treatment, while eleven genes (*DlWRKY*2, 6, 12, 13, 14, 19, 29, 33, 34, 40, and 50) showed no significant differential expression in response to the given treatments. The SA treatment induced the expression of the 22 *DlWRKY* genes (*DlWRKY*1, 3, 5, 8, 10, 15, 16, 18, 23, 26, 27, 32, 36, 38, 42, 43, 45, 46, 48, 51, 54, and 55) but reduced the expression of five *DlWRKY* genes (*DlWRKY*9, 20, 24, 25, and 41). Fifteen *DlWRKY* genes (*DlWRKY*1, 3, 4, 8, 10, 16, 21, 32, 38, 39, 44, 45, 51, 53, and 55) were up-regulated, and three (*DlWRKY*20, 25, and 41) were down-regulated by MeJA treatment. For heat treatment, 11 (*DlWRKY*4, 9, 20, 27, 28, 35, 37, 39, 44, 49, and 52) and 4 (*DlWRKY*5, 8, 16, and 51) genes were down-regulated or up-regulated, respectively. A total of 17 *DlWRKY* (*DlWRKY*5, 7, 9, 17, 18, 20, 23, 25, 26, 31, 37, 39, 41, 42, 47, 51, and 54) genes showed up-regulated expressions, and no genes were down-regulated by cold treatment. Under the drought treatment, 20 (*DlWRKY*1, 4, 5, 8, 10, 11, 15, 16, 17, 21, 22, 25, 26, 27, 28, 30, 36, 45, 48, and 51) and 7 *DlWRKY* genes (*DlWRKY*9, 35, 37, 41, 44, 49, and 54) were up-regulated or down-regulated, respectively. Eighteen (*DlWRKY*1, 4, 5, 8, 10, 11, 15, 16, 18, 21, 23, 32, 36, 38, 42, 45, 48, and 51) and five *DlWRKY* genes (*DlWRKY*9, 20, 24, 37, and 41) were up-regulated or down-regulated, respectively, under high salinity treatment.

### 2.7. Analysis Related Cis-Elements in the Candidate DlWRKY Genes

**Figure 7:**
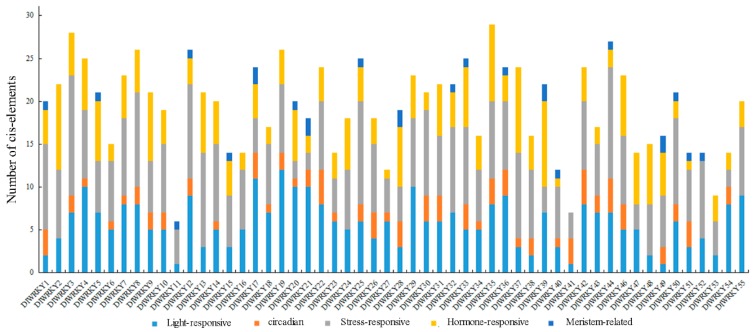
The predicted *cis*-elements in the promoter of the *DlWRKY* genes. The 1.5 kb sequences of 55 *DlWRKY* genes were analyzed with the PlantCARE software.

To analyze the potential function of *DlWRKY* genes in response to various responses, the *cis*-elements in the promoter region of the *DlWRKY* genes were further analyzed. Among these 55 genes, 54 genes could perform *cis*-elements analysis except *DlWRKY*45, which only contain 270 promoter bases. All the *DlWRKY* genes shared the light-responsive boxes and stress-responsive boxes in their promoter. Hormone-related *cis*-elements, such as AuxRR-core, TCA-element, CGTCA-motif, GARE-motif, P-box, and ERE (Ethylene-responsive element), existed in the promoter of all *DlWRKY* genes except *DlWRKY*11, *DlWRKY*41, and *DlWRKY*52. Additionally, circadian-related *cis*-elements were found in the promoter of 39 *DlWRKY* genes and Meristem-related *cis*-elements were only presented in the promoter of 20 *DlWRKY* genes (Figure 7, Tables S5 and S6).

## 3. Discussion

The WRKY proteins, an important transcription factor superfamily which is involved in plant development and stress responses, have been widely detected in various organisms from single-celled green algae to monocots and dicots [15]. Recently, the successful genome sequencing of longan makes it possible to analyze WRKY TFs at the whole-genome level [37]. The present study is the first to identify and characterize WRKY proteins from whole-genome sequences of longan.

In this study, we identified 59 candidate WRKY genes in the longan genome (471.88 Mb) using the HMM and Blastn search methods. These genes included 58 *DlWRKYs*, which were also found by Lin et al. [37], and one gene *Dlo_*022548.1 (*DlWRKY*36) found in our study. Finally, after the WRKY domain scanning and sequence alignment, 55 *DlWRKY* genes were determined in the longan genome (Table 1). The number of *WRKY* genes in longan was similar to those found in grape (59 *VvWRKYs*), whose genome size is 487 Mb, which is similar to that of the longan genome [29]. However, the size of the WRKY family in longan is smaller than that in *A. thaliana* (72), *Oryza sativa* ssp. *Indica* (102), and the common bean (88), although their genome sizes are similar (*O*. *sativa* ssp. *Indica*, 466 Mb; common bean, 587 Mb) or even smaller (*A. thaliana*, 119 Mb) than the longan genome size (Table S7) [28,38,39]. Therefore, the number of WRKY family members is not necessarily correlated with the genome size. Previous studies showed that the only group I *WRKYs* are present in green algae and all *WRKY* genes originated from the group I C-terminal WRKY domains, whereas group II members were evolved in the common ancestor of land plants, and Group III members emerged in the common ancestor of seed plants [15]. In addition, as a newly defined and the most dynamic group with many duplication events, the differences in the number of *WRKY* genes in Group III are the primary cause of the sizes of *WRKY* gene families [40]. In the present study, the differences in the number of *WRKY* genes between longan and *Arabidopsis* mainly existed in groups IIc and III, indicating that the group IIc and III *WRKY* genes may play important roles in the functional evolution of *DlWRKYs*.

According to the classification scheme for the WRKY family of Eulgem et al. [41], the DlWRKY proteins were divided into three distinct clusters: groups I, II, and III. Group II proteins were further divided into five distinct groups: a–e (Figure 1 and Table 1). In addition, subgroup IIc contained the largest number of WRKY proteins. These results were consistent with the results observed in other species [28,29,42,43,44]. The WRKY motif was fairly conserved in longan WRKY proteins, and three variants of this motif were observed. All the DlWRKYs, except DlWRKY19 and DlWRKY47, possessed WRKYGQK. DlWRKY19, which belonged to subgroup IIc, possessed WRKYGKK. DlWRKY19, which belonged to subgroup III, possessed WKKYRQK. In the common bean, the variants WRKYGKK, WRKYGEK, WKKYEDK, and WKKYCEDK are mainly observed in subgroup IIc [28]; in mulberry, WRKYGKK is detected in subgroup IIb [27]. Moreover, in rice, nine variants, most of which belong to groups III and IIc, are observed [45]. Previous studies showed that these variations of the WRKYGQK motif might change the DNA binding specificities of downstream target genes, and WRKY genes with the variations of the WRKYGQK motif may recognize binding sequences other than the W-box element ((C/T)TGAC(C/T)) [15]. Hence, the result suggested that DlWRKY19 and DlWRKY47 may possess different binding specificities and functions from those of other DlWRKY proteins.

WRKY family genes play important roles in diverse plant development and shown a tissue-specific expression in many plant species [15,40]. For example, *AtWRKY*75 exerts a negative effect on root hair development [46]. *SUSIBA*2 [47] and *MINISEED*3 [48] play roles in the regulation of seed development. In grape, nearly half of the 59 *VvWRKY* genes show no significant organ/tissue-related differences in expression, and some clear spatial differences are noted [29]. In mulberry, 13 *WRKY* genes exhibit the highest expression in the *Morus notabilis* root tissue. A maximum of 25 *WRKYs* show the highest expression in the bark tissue, and 10 WRKY genes display the highest expression in other stages [27]. In the present study, the expression profiles of 55 longan *WRKY* genes in nine longan tissues were ascertained by RNA-seq analysis (Figure 4). The results demonstrated variation in the expression pattern of *DlWRKY* genes. In total, 25 *DlWRKY* genes (*DlWRKY*1, 2, 3, 5, 6, 8, 9, 13, 14, 23, 24, 28, 30, 32, 35, 37, 38, 39, 44, 49, 50, 52, 53, and 54) were highly expressed in at least six longan tissues. As highly expressed genes usually play important roles in plant development [44], we concluded that the 25 highly expressed *DlWRKY* genes might be important regulatory factors in longan development. It was found that group I and group IId *WRKY* genes are ancestral to other *WRKY* genes in plants or algae and are more likely to be constitutively expressed in different tissues [15,40]. For instance, most of the highly expressed *SiWRKY* genes belonged to group I and IId [40]. Consistent with these studies, in the present study, most of the members of groups I (9 of 11) and IId (4 of 6) were the highly expressed gene. In contrast, 12 *DlWRKY* genes were expressed at low levels in all tested tissues and these minimally expressed *DlWRKY* genes were distributed in almost all the *WRKY* gene subgroups except for IId. Meanwhile, six *DlWRKY* genes were preferential accumulation in no more than three tissues, implying that these genes might play crucial roles during the development of specific organs. Additionally, these specifically or minimally expressed *DlWRKY* genes could be induced under environment stimuli. For example, *DlWRKY*10, 22, 41, and 47 were not detected in leaves under normal conditions, but they were induced by different abiotic stresses (Figure 6). Similar results were also found in other studies [15,40,49].

Perpetual flowering is a crucial trait for fruit trees as it enlarges the production period [50]. To date, the genetic control of PF has been deciphered in several model plants. For example, In *Arabidopsis*, the PF trait is controlled by *PERPETUAL FLOWERING 1* (*PEP1*), an orthologue of the FLC floral repressor [51]. In the diploid strawberry and rose, the PF trait is due to a mutation in the orthologue of the *TERMINAL FLOWER* 1 (*TFL*1) floral repressor [50,52]. Recent studies showed that the PF trait of some cultivated strawberries is genetically controlled by the major *FaPFRU* locus, which is non-orthologous to *TFL*1 [53,54]. However, the multi-year delay in the onset of flowering and the long juvenile phase hampers the research of PF traits in perennials, such as longan. Although WRKY TFs regulate various plant developments, only a few data are available on whether WRKY TFs are involved in the flowering time regulation. Meanwhile, as a kind of TF, WRKY genes regulated plant flowering by being directly active or inhibiting the downstream target gene. For example, promoter sequences of *FT*, *LFY*, and *AP1* harbor W-boxes (TTTGACT/C); *AtWRKY*71 affects the flowering time of plants by directly regulating these genes [16]. In our study, all the 55 *DlWRKY* genes were constructively expressed in the three test flower induction process of the “SX” longan, while 18 *DlWRKY* genes showed a specific expression in the “SJ” longan (Figure 5a). This result indicated that these 18 *DlWRKY* genes may specifically be involved in the flower induction of “SJ”. In summary, we proposed that these 18 *DlWRKY* genes may participate in the forming of the longan PF habit, which further studies are required to verify the function of these genes.

WRKY genes play crucial roles in the response to abiotic and biotic stress-induced defense signaling pathways [15]. Numerous studies have demonstrated that WRKY genes are expressed strongly and rapidly in response to particular abiotic stresses [15,22,29,40,52]. Consistent with these previous studies, our study showed that 44 *DlWRKY* genes (80%) showed up- or down-regulated expression in at least one tested treatment (Figure 6 and Figure S1), thereby highlighting the extensive involvement of *WRKY* genes in environmental adaptation. SA, JA, and Eth play important roles in biotic and abiotic stresses [55]. Many *WRKYs*, such as *AtWRKY*28, *AtWRKY*46, *AtWRKY*70, and *AtWRKY*54, play an important role in SA- and JA-dependent defense signaling pathways [53,56,57]. In the present study, 27 and 18 *DlWRKY* genes were up- or down-regulated by SA and MeJA treatment, respectively. For example, *DlWRKY*25, the orthologue of *AtWRKY*70 and *AtWRKY*54, was regulated by the SA and JA treatments. *AtWRKY*25 and *AtWRKY*33 regulate plant adaptation to salinity stress through an interaction with their upstream or downstream target genes [58]; their orthologue *DlWRKY*8 in longan was regulated by SA, JA, heat, drought, and salinity. In grape [29], *VvWRKY*42 and its orthologue *DlWRKY*11 in our study were up-regulated by salt treatment. Furthermore, we observed same orthologous genes with different expression patterns under stress treatment. *DlWRKY*44 was down-regulated under drought, and its orthologous gene *VvWRKY*35 was up-regulated under this stress treatment. *DlWRKY*19 showed no significant differential expression in response to salinity, and its orthologous gene *VvWRKY*25 was up-regulated [29]. We speculate that these orthologous genes may be involved in the different signaling pathways in different species. Additionally, only one gene (*DlWRKY*52) was significantly highly expressed under all abiotic stresses. These results indicated that the different *DlWRKYs* played different roles in regulating stress response and that further investigation of the functions of these *DlWRKY* genes is necessary. Differential responses of several *WRKYs* are regulated by the presence of *cis*-elements in their promoter region [27,40,49]. For example, *Morus*013217, which contains three LTREs in its promoter regions showed a strong response to cold stress [27]. Similar results were also found in our study. For instance, four HSEs were found in the promoter regions of *DlWRKY*2, which showed a strong response to heat stress. *DlWRKY*36, *DlWRKY*46, and *DlWRKY*48 showed responsiveness to SA treatment and their expressions were all up-regulated, and more than two TCA-elements were found in their promoters. While the *DlWRKY*11 and *DlWRKY*52 hormone-related *cis*-elements existed in their promoter, they showed no response to the SA or MeJA treatments (Figure 6 and Table S6). Thus, these *cis*-elements could provide more evidence of the DlWRKY genes in response to different stresses or hormonal signaling.

## 4. Materials

### 4.1. Identification of Longan WRKY Genes

Longan whole-genome sequences, transcript data, and proteins were downloaded from the NCBI Sequence Read Archive (SRA315202) or ftp://climb.genomics.cn/pub/10.5524/100001_101000/100276/ [37]. The HMM profile of the WRKY DNA binding domain (PF03106) which was extracted from the Pfam database (http://pfam.sanger.ac.uk/) was used to obtain the potential members of the longan WRKY genes [59] and used to search the putative WRKY genes from the longan genome with HMMER 3.0 (http://hmmer.janelia.org/) with the default parameters and 0.01 as the cutoff value. Then, all non-redundant longan WRKY protein sequences were selected and the domain was conserved using Simple Modular Architecture Research Tool (http://smart.emblheidelberg.de/) [60].

### 4.2. Sequence Alignment, Phylogenetic Analysis, and Cis-Elements in the Promoters

The 72 *Arabidopsis* and 59 grape WRKY proteins described previously [29,38] were obtained from TAIR (http://www.arabidopsis.org/) and NCBI (http://www.ncbi. nlm.nih.gov/), respectively. By using Clustal X version 1.83, the WRKY protein sequences of *Arabidopsis* and longan were aligned for phylogenetic analysis. Based on this alignment, a bootstrapped ML (Maximum Likelihood) tree was constructed using MEGA (version 6.0) with the bootstrap test replicated 1000 times [61]. To assess the phylogenetic relationships among the members of the longan *WRKY* gene family, a phylogenetic tree was prepared according to the alignment of only the longan proteins. All DlWRKY transcription factors were classified into subgroups based on their structural features and evolutionary relationships. The 1500-bp sequences upstream of the start codon of the candidate *DlWRKY* genes were extracted from the longan genome sequences. The PlantCARE software (http://bioinformatics.psb.ugent.be/webtools/plantcare/html/) was used for searching the cis-acting elements [62].

### 4.3. Protein Feature Analysis

The ExPASy online tools (http://expasy.org/tools/) [63] were used to calculate the MW, the number of amino acids, the ORF, ORF length, and isoelectric point (pI) of DlWRKY proteins. The arrangements and the intron and exon junctions of the *DlWRKY* genes were analyzed by the GSDS, version 2.0 [64]. MEME (http://meme.sdsc.edu/meme/cgi-bin/meme.cgi) [65] was used to analyze the conserved motifs of the DlWRKY proteins with the following optimized parameters: any number of repetitions; maximum number of motifs: 15; and the optimum width of each motif: between 6 and 50 residues.

### 4.4. Expression Analysis of Longan WRKY Genes in Various Tissues and Different Flowering Stages

The RNA-seq data for analyzing the expression patterns of *WRKY* genes in different longan tissues were downloaded from the NCBI Sequence Read Archive (GSE84467). Three pairs of nine-year-old “SJ” and “SX” *D. longan* trees which displayed opposite flowering phenotype were used for comparative expression analysis of *DlWRKY* during floral induction. All those trees were grown at an experimental orchard in the South Subtropical Crops Research Institute of the Chinese Academy of Tropical Agricultural Science in Zhanjiang (110°16′ E, 21°10′ N), China. Three different kinds of apical buds, including the dormant stage (T1), the emergence of floral primordia stage (T2), and the floral organ formation stage (T3) of “SJ” and “SX”, were used in this study. The samples obtained for the T1, T2, and T3 in “SJ” and “SX” were collected on 20 November 2016, 24 December 2016, and 1 January 2017, respectively. For each sample, we used three biological replicates from three different trees. Each biological replicate contained the mixed buds which were collected from the four cardinal directions of each tree. All samples were collected from 10:00 am to 12:00 am and were frozen immediately in liquid nitrogen and stored at −80 °C. According to the manufacturer’s instructions, the total RNA was extracted by using the quick RNA Isolation Kit (Hua Yue Yang Bio Co., Ltd., Beijing, China) and the genomic DNA residues were removed during RNA extraction. We used an Agilent 2100 Bioanalyzer (Agilent, Santa Clara, CA, USA) to test the RNA concentration and the quality of each sample. The RNA quality was also confirmed by RNase free agarose gel electrophoresis. The RNA-seq experiment was performed as described by our previous study [66]. The RNA-seq data were uploaded to the NCBI Sequence Read Archive (SRS2241241, SRS2241242, SRS2241243, SRS2241244, SRS2241245, SRS2241246, SRS2241247, SRS2241248, SRS2241249, SRS2241250, SRS2241251, SRS2241252, SRS2241253, SRS2241254, SRS2241255, SRS2241256, SRS2241257, and SRS2241258). The fragments per kilobase of the exon model per million mapped values (FPKM) were log_10_-transformed, and heat maps with hierarchical clustering were exhibited using the software Mev4.9.0 [67].

### 4.5. Stress and Hormonal Treatments and Expression Profiling Using qRT-PCR

Twenty-seven one-year-old uniform grafted seedlings of “SJ”, obtained from the South Subtropical Crops Research Institute of the Chinese Academy of Tropical Agricultural Science in Zhanjiang (110°16′E, 21°10′N) were used for stress and hormonal treatments. For hormone treatments, three seedlings were treated with methyl jasmonate (MeJA) or SA solution (100 μM) for 4 h at 28 °C, respectively. Meanwhile, three seedlings sprayed with water were used as a control. For heat and cold stresses, three samples were grown at 42 or 0 °C for 4 h, respectively, and three samples grown at 28 °C were used as a control. All the treatments were performed in a greenhouse. Six leaves were collected from each seedling and all samples were immediately frozen in liquid nitrogen and stored at −80 °C for expression analysis.

According to the manufacturer’s instructions, the total RNA was obtained by using the SuperFast RNA extraction kit (Hua Yue Yang Bio Co.). The first-strand cDNA was synthesized by reverse transcription of the total RNA (500 ng) using PrimeScriptRTase (TaKaRa Biotechnology, Dalian, China). Gene-specific primers were designed according to the *DlWRKY* gene sequences using Primer Premier 5.0 and checked using Blastn in NCBI (Table S8). In addition, the longan *Actin*1 gene (Dlo_028674) was used as an internal control for normalization. qRT-PCR was conducted using the LightCycler^®^ 480 Real-Time PCR System (Roche, Germany) and SYBR Green II PCR Master Mix (Takara, Dalian, China). The amplification program was as follows: 95 °C for 5 min, followed by 40 cycles of 95 °C for 15 s, and 60 °C for 1 min. Each reaction was performed in three replicates. The relative expression levels of the candidate genes were calculated by the 2^–∆∆*C*t^ method. The analysis included cDNA from the three biological samples for each tissue, and all the reactions were run in triplicates. In the comparative expression analysis of the *DlWRKY* genes, genes that were up- or down-regulated by at least two-fold were considered differentially expressed.

## 5. Conclusions

It is essential to systematically analyze the function of transcription factors (TFs), since these genes can regulate the expression of many others, resulting in deep physiological modifications. Although *WRKY* genes have been identified in many other species, the information of longan *WRKY* is still unknown. In the present study, we conducted a genome-wide identification and analysis of the *WRKY* genes in longan. A total of 55 *DlWRKY* genes were identified in the longan genome. Phylogenetic analysis indicated that these 55 *DlWRKYs* could be divided into seven groups. An RNA-seq-based analysis showed that several of the identified *WRKY* genes may play various roles in the development of longan tissues. In addition, comparative expression analysis revealed that 18 *DlWRKY* genes might have participated in the regulation of longan flowering. Our RNA-seq, qRT-PCR, and promoter analyses revealed the gene expression profiles and implied that the response to different stress or hormonal signaling of some *DlWRKY* may be due to the cis-elements in their promoters. In summary, our results will facilitate further studies into the role of *DlWRKY* genes in response to abiotic stresses and the development of molecular breeding programs to enhance abiotic stress tolerance and increase yield in longans.

## Figures and Tables

**Table 1:** The information of the *DlWRKY* gene family.

Gene Name	Gene Locus ID	Location	ORF (bp)	Size (aa)	PI	MW (KDa)	Intron	Full Length
*DlWRKY*1	Dlo_000299.1	scaffold1:3145979:3147233	1071	356	9.63	38.76	2	1255
*DlWRKY*2	Dlo_026119.1	scaffold6:875263:878308	894	297	6.26	32.31	2	3046
*DlWRKY*3	Dlo_026149.1	scaffold6:1127159:1130416	1596	532	7.26	57.64	3	3258
*DlWRKY*4	Dlo_026267.1	scaffold6:2195842:2200859	1815	605	6.66	66.08	4	5018
*DlWRKY*5	Dlo_030713.1	scaffold8:184175:186167	1059	353	5.63	39.46	2	1993
*DlWRKY*6	Dlo_002181.1	scaffold11:1861336:1864296	1767	589	7.23	64.37	4	2961
*DlWRKY*7	Dlo_012455.1	scaffold23:1107291:1111499	1914	638	6.75	69.01	5	4209
*DlWRKY*8	Dlo_013053.2	scaffold24:1070557:1072933	1668	556	6.52	61.46	4	2377
*DlWRKY*9	Dlo_015501.2	scaffold29:1782026:1783019	762	254	8.99	28.30	4	994
*DlWRKY*10	dlo_037126.1	scaffold29:1793158:1794294	684	228	9.02	25.58	2	1146
*DlWRKY*11	Dlo_016404.1	scaffold31:1522675:1524675	1026	342	5.60	38.86	2	2001
*DlWRKY*12	Dlo_019125.1	scaffold38:1882835:1883726	480	160	5.16	18.38	2	892
*DlWRKY*13	Dlo_023965.1	scaffold53:1206068:1207919	1035	345	9.77	38.51	2	1852
*DlWRKY*14	Dlo_028963.1	scaffold71:878665:881164	1613	471	8.87	51.80	3	2500
*DlWRKY*15	Dlo_031097.1	scaffold81:147303:148636	972	324	6.33	35.30	2	1334
*DlWRKY*16	Dlo_033905.1	scaffold98:272537:275137	1419	473	5.82	51.20	2	2601
*DlWRKY*17	Dlo_001368.1	scaffold105:274029:278833	1425	475	6.10	52.14	4	4805
*DlWRKY*18	Dlo_003898.1	scaffold124:605265:607367	1053	351	9.04	39.34	1	2103
*DlWRKY*19	Dlo_003928.1	scaffold124:1058067:1061659	633	211	6.37	23.26	2	3593
*DlWRKY*20	Dlo_004435.1	scaffold129:429868:432959	1644	548	7.41	59.78	5	3092
*DlWRKY*21	Dlo_008095.1	scaffold167:682922:683969	714	238	5.14	26.59	2	1048
*DlWRKY*22	Dlo_008126.1	scaffold168:307774:310141	1245	415	4.62	44.95	1	2368
*DlWRKY*23	Dlo_008610.1	scaffold176:75022:76492	1023	341	8.62	38.08	4	1471
*DlWRKY*24	Dlo_009865.1	scaffold192:233555:234849	1071	357	5.50	39.25	2	1295
*DlWRKY*25	Dlo_011410.1	scaffold213:248908:250725	1038	346	5.93	38.65	2	1818
*DlWRKY*26	Dlo_011411.1	scaffold213:253855:257080	1122	374	6.00	40.22	2	3226
*DlWRKY*27	Dlo_012276.1	scaffold229:13116:15182	1005	335	7.16	37.09	2	2067
*DlWRKY*28	Dlo_012878.1	scaffold238:352167:354143	1527	509	5.89	55.49	3	1977
*DlWRKY*29	Dlo_013340.1	scaffold245:258019:261130	696	232	8.95	26.57	2	3112
*DlWRKY*30	Dlo_013413.1	scaffold247:267246:270528	2238	746	5.59	80.60	4	3283
*DlWRKY*31	Dlo_014324.1	scaffold266:341214:343700	663	221	7.71	25.38	3	2487
*DlWRKY*32	Dlo_015139.1	scaffold286:162902:164294	1059	353	6.32	38.46	2	1393
*DlWRKY*33	Dlo_015144.1	scaffold286:195837:198196	615	205	9.03	23.13	1	2360
*DlWRKY*34	Dlo_015224.1	scaffold287:217068:218497	480	160	9.54	18.10	1	1430
*DlWRKY*35	Dlo_016828.1	scaffold322:63655:67562	1326	442	9.62	48.27	4	3908
*DlWRKY*36	Dlo_022548.1	scaffold487:170363:175747	3813	1271	5.15	143.77	5	5385
*DlWRKY*37	Dlo_023098.1	scaffold502:191885:193351	1056	352	9.46	38.46	2	1467
*DlWRKY*38	Dlo_023764.1	scaffold524:170088:173717	1533	511	8.66	55.75	3	3630
*DlWRKY*39	Dlo_025188.1	scaffold568:191129:193577	1530	510	8.26	55.75	5	2449
*DlWRKY*40	Dlo_025974.1	scaffold597:89062:90386	1110	370	5.07	40.99	2	1325
*DlWRKY*41	Dlo_026484.1	scaffold607:21585:23785	1218	406	6.06	45.38	4	2201
*DlWRKY*42	Dlo_027244.2	scaffold640:85638:89083	2298	766	5.15	83.59	4	3446
*DlWRKY*43	Dlo_027361.1	scaffold648:191661:193182	969	323	9.14	36.57	2	1522
*DlWRKY*44	Dlo_027614.1	scaffold657:107511:111179	1521	507	5.55	54.88	4	3669
*DlWRKY*45	Dlo_029034.1	scaffold711:179562:181398	1539	513	8.27	55.15	2	1837
*DlWRKY*46	Dlo_029939.1	scaffold757:33093:37889	1710	570	6.38	61.42	5	4797
*DlWRKY*47	Dlo_031466.1	scaffold829:42224:45497	1023	341	7.20	7.71	4	3274
*DlWRKY*48	Dlo_031469.1	scaffold829:58277:59797	990	330	9.06	36.21	3	1521
*DlWRKY*49	Dlo_031936.1	scaffold858:266912:269300	588	196	9.46	22.05	1	2389
*DlWRKY*50	Dlo_032595.1	scaffold896:87649:89238	1185	395	6.67	43.04	2	1590
*DlWRKY*51	Dlo_033966.1	scaffold980:88739:90132	933	311	5.14	34.86	2	1394
*DlWRKY*52	Dlo_001658.1	scaffold1077:66972:68290	918	306	6.26	33.96	4	1319
*DlWRKY*53	Dlo_002663.1	scaffold1135:95286:97669	1929	643	5.73	70.07	4	2384
*DlWRKY*54	Dlo_004749.1	scaffold1314:73982:75144	795	265	5.24	30.27	2	1163
*DlWRKY*55	Dlo_010873.1	scaffold2042:2013:3910	1023	341	9.42	37.97	3	1898

## Supplementary Materials

Supplementary materials can be found at http://www.mdpi.com/1422-0067/19/8/2169/s1. The following are available online. Table S1. The candidate *WRKY* genes and their protein structure found in the longan genome. Table S2. The information for each motif of DlWRKYs. Table S3. FPKM values of *DlWRKY* genes in nine tissues of longan. Table S4. FPKM values of *DlWRKY* genes in the three flower induction stages of “SJ” and “SX” longan species. The red color indicates the genes which showed down-regulated expression; the blue color indicates the genes which showed up-regulated expression; and the green color indicates the genes that showed an up-regulated expression in the first two stages and a down-regulated expression in the third stage. Table S5. Details of the *cis*-elements identified in this study. Table S6. Predicted *cis*-elements in the promoter of the *DlWRKY* genes. Table S7. The WRKY gene number and genome size of different species. Table S8. Primers used in quantitative RT-PCR of *DlWRKY* genes. Figure S1. Expression patterns of selected *DlWRKY* genes which have no significant difference under various hormonal and abiotic stresses. The x-axis indicates various treatments, and the y-axis indicates the relative expression level. Error bars were obtained from three independent biological replicates.

## Abbreviation

HMM	Hidden Markov model
NJ	Neighbor-joining
GSDS	Gene Structure Display Server
MW	The molecular weight
ORF	Open reading frame
pI	Isoelectric point
NCBI	National Center of Biotechnology Information
qRT-PCR	Quantitative real-time reverse transcription polymerase chain reaction
RNA-seq	RNA sequencing
SA	Salicylic acid
JA	Jasmonic acid
