# Supplementary figures and images for: Identification of WRKY Gene Family from Dimocarpus longan and Its Expression Analysis during Flower Induction and Abiotic Stress Responses
Source: Int J Mol Sci. 2018 Jul 25;19(8):2169. doi: 10.3390/ijms19082169 (PMC6121330; doi:10.3390/ijms19082169)

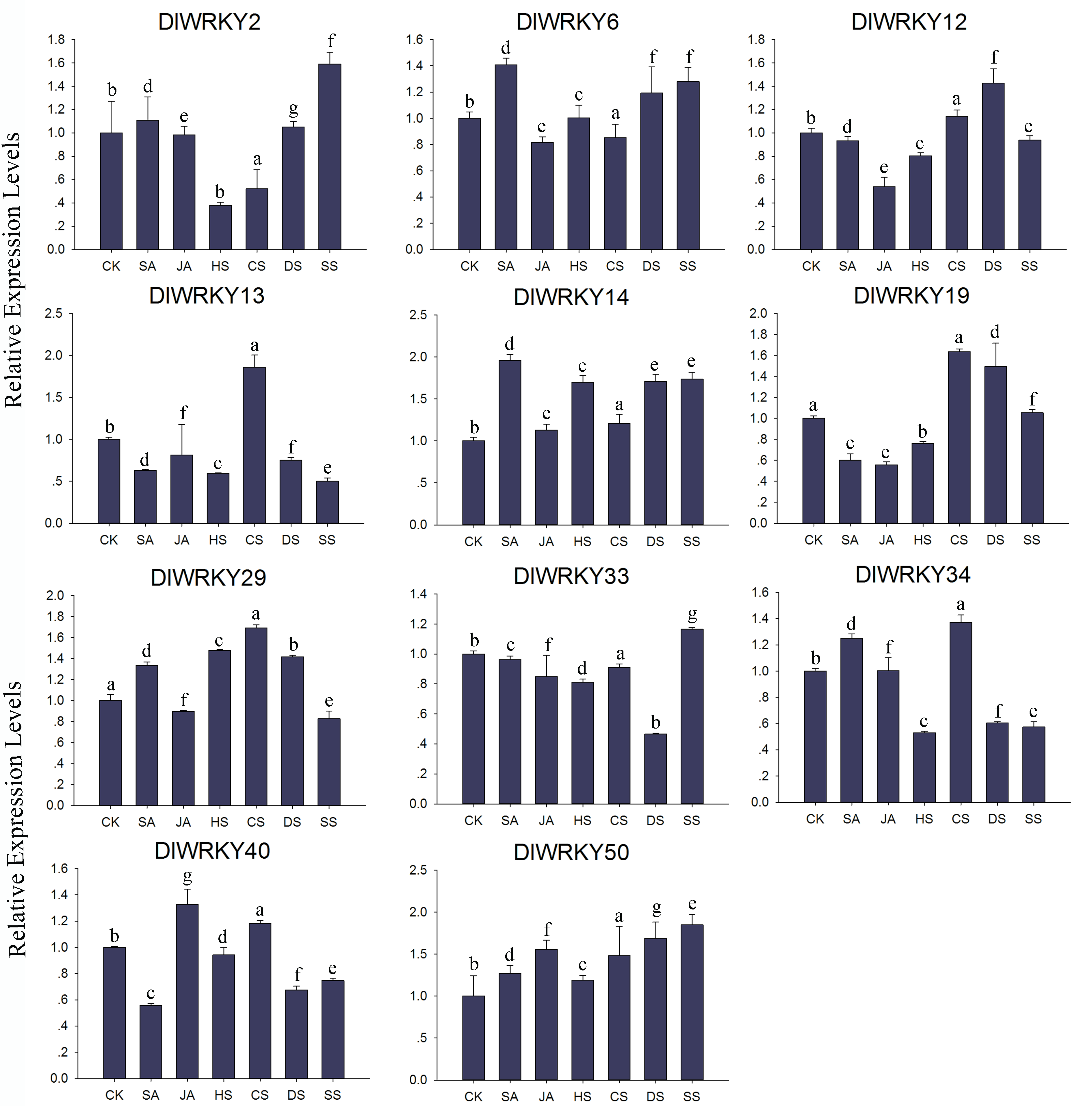

Supplement: Supplementary file 1 [file ijms-19-02169-s001.zip › ijms-319863-SI/Supplementary Figure S1.tif]
